# Specter of Epidemic Typhus

**DOI:** 10.3201/eid2904.AC2904

**Published:** 2023-04

**Authors:** Byron Breedlove

**Affiliations:** Centers for Disease Control and Prevention, Atlanta, Georgia, USA

**Keywords:** epidemic typhus, typhus fevers, scrub typhus, murine typhus, epidemic typhus, Rickettsia prowazekii, typhus louse, lice, Charles-Jules-Henri Nicolle, Henrique da Rocha Lima, O. Grin, The Typhus Louse Shaking Hands with Death, specter of epidemic typhus, public health posters, death, public health, bacteria, about the cover, art and science, insect vectors, zoonoses

**Figure:**
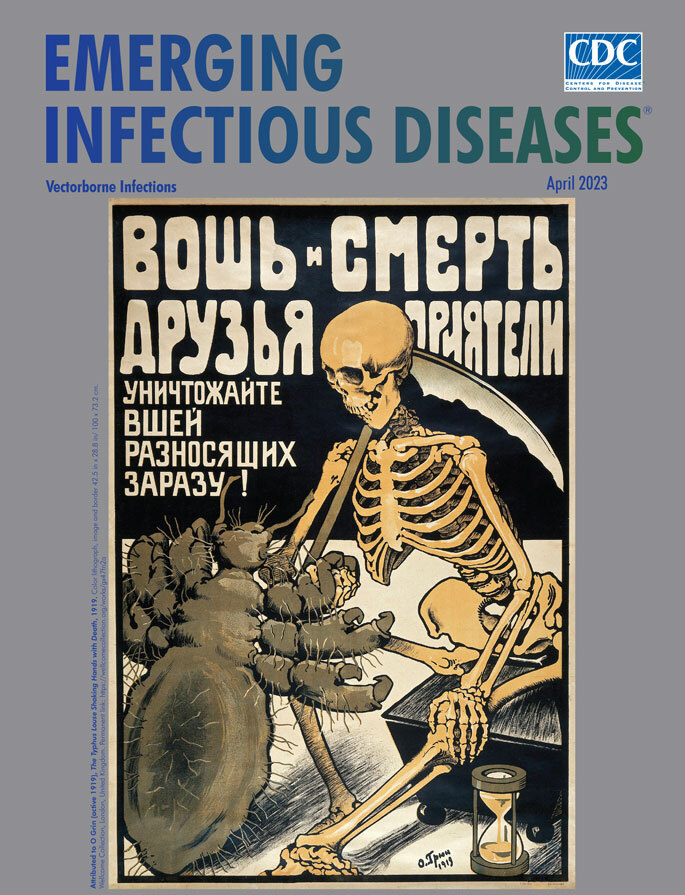
**Attributed to O. Grin (active 1919), *The Typhus Louse Shaking Hands with Death,* 1919.** Color lithograph, image and border 42.5 in x 28.8 in/100 × 73.2 cm. Wellcome Collection, London, United Kingdom. Permanent link: https://wellcomecollection.org/works/gx47fn2a

Typhus fevers—including scrub typhus, murine typhus, and epidemic typhus—are vectorborne rickettsial diseases spread to humans by chiggers, fleas, and lice, respectively. Epidemic typhus, sometimes called louse-borne typhus, is caused by the bacterium Rickettsia prowazekii*, and* this form of typhus is transmitted to humans by the body louse *Pediculus humanus humanus.*

Though now considered an uncommon disease, epidemic typhus outbreaks resulted in millions of deaths during previous centuries in Europe, Mexico, South America, and Central America. Such outbreaks were prevalent among people living in poverty, displaced and homeless populations, prisoners, and military troops. According to infectious disease researchers Emmanouil Angelakis, Yassina Bechah, and Didier Raoult, “Epidemic typhus has accompanied disasters that impact humanity and has arguably determined the outcome of more wars than have soldiers and generals.” Now, new concerns exist about possible outbreaks of epidemic typhus in war-torn areas, such as parts of Ukraine.

In 1909, French physician and microbiologist Charles Nicolle discovered the mode of transmission for epidemic typhus. Myron Schultz and David Morens wrote that Nicolle “was well aware of the clinical presentation of typhus—its triad of fever, rash, and stupor—and of its link to poverty” and that he “reasoned that lice on patients’ clothes were most likely the vectors.” Medical historian K. David Patterson noted that “the Russian term *platyanaya vosh'*, clothes louse, is more accurate that the English term” body louse because these insects live in the inner folds of clothing, from where they make frequent forays onto their host’s skin for blood meals. Although Nicolle did not determine the infectious agent that causes epidemic typhus, his discovery of the vector proved instrumental in helping control its outbreaks. 

In 1916, Brazilian physician Henrique da Rocha Lima established that the disease was caused by a bacterium he named Rickettsia prowazekii. That name honors the legacy of American pathologist Howard T. Ricketts and Czech bacteriologist Stanislaus Joseph Mathias von Prowazek, Rocha Lima’s colleague, both of whom contracted fatal cases of typhus during their research. In 1920, S. Burt Wolbach conducted ensuing research that confirmed that lice were the vectors for R. prowazekii.

*Patterson wrote, “Typhus was a major health problem in late 19th and 20th century Russia, and great epidemics flared up whenever war or famine produced hardship and massive population movements. Major typhus epidemics took place late in World War I and in the years of civil war following the Bolshevik Revolution. Typhus claimed some 2 to 3 million lives from 1918 to 1922.”*

Reaching at-risk populations in crowded cities and remote communities with information about diseases, including typhus, was challenging. Visually arresting public health posters directed at both civilian and military populations provided a relatively inexpensive way to reach large numbers of people, many of whom were illiterate. Communications professor David Serlin noted, “Although the war was hardly the cause of the epidemical public health poster, the authoritarian conditions of wartime and the extensive use of posters for recruiting were favorable to it.” 

This month’s cover image shows a Russian public health poster from 1919, the third year of the Russian civil war, and the year Vladimir Lenin declared that *“*Either the louse will defeat socialism, or socialism will defeat the lice*.”* It is cataloged in the Wellcome Collection with the title *The typhus louse shaking hands with Death*. 

*Embodied as a leering* skeleton, *Death* sits on a black bench, a well-used scythe slung over its shoulder, an hourglass resting at its feet, and empty eye sockets fixated on a grotesque, engorged, enlarged louse. Death clasps the front leg of the louse as the pair seal a mortal contract, visually communicating the peril posed by louse infestations and typhus infection. Featured prominently along the top of the poster in Cyrillic script (translated here into English) is the exhortation, “The Louse and Death are friends and comrades. Kill all lice that carry infection.” No accompanying information sheds details about O. Grin, credited with creating this poster, nor has anything come to light after sleuthing via internet searches for this moniker. 

Outbreaks of epidemic typhus still occur in the Andes regions of South America and some parts of Africa. Sporadic cases are reported in the United States when people are exposed to flying squirrels or their nests. However, conflict and disasters raise the specter of reemergence of epidemic typhus, and it is still considered a public health threat. Modern medicine provides diagnostic tools and the antibiotic doxycycline to mitigate *R. prowazekii* infection outbreaks, but early detection remains essential.
